# A Case of Tubal Pregnancy

**Published:** 1884-12

**Authors:** 


### A Case of Tubal Pregnancy.

Dr. Hun, of Albany, reports in the *American Journal of the Medical Sciences*, for July, 1884, a case of tubal pregnancy on the right side, the corpus cuteum being in the left ovary. The sac raptured about the twenty-fifth day, followed by death of the patient. There were no local causes found to account for lodging of the ovum in the tube, such as occlusion of the tube by pressure of a tumor, inflammatory adhesions, or uterine displacement. If the corpus cuteum found in the left ovary were really the remains of the Graafian follicle, from which this impregnated ovum was discharged, the reason why it was not taken up by the left fallopian tube would be of interest.

